# Gesundheitsförderung auf dem Campus – Wie es Studierenden geht und was sie sich wünschen: Ergebnisse einer Befragung unter Studierenden der Erziehungswissenschaft und der Humanmedizin an der Universität Augsburg

**DOI:** 10.1007/s11553-023-01051-6

**Published:** 2023-06-06

**Authors:** Carolin Rolle, Petra Götte, Thomas Rotthoff

**Affiliations:** 1 Lehrstuhl für Medizindidaktik und Ausbildungsforschung (DEMEDA), Medizinische Fakultät, Universität Augsburg, Universitätsstr. 2, 86159 Augsburg, Deutschland; 2 Lehrstuhl für Pädagogik, Philosophisch-Sozialwissenschaftliche Fakultät, Universität Augsburg, Universitätsstr. 2, 86159 Augsburg, Deutschland

**Keywords:** Gesundheitszustand, Prävention, Bedarf, Psychische Gesundheit, COVID-19, Health status, Prevention, Demand, Mental health, COVID-19

## Abstract

**Hintergrund:**

Studierende in Deutschland weisen seltener einen guten subjektiven Gesundheitszustand und häufiger eine depressive Symptomatik auf als die 18- bis 29-jährige Allgemeinbevölkerung.

**Ziel und Fragestellung:**

Ziel der Befragung ist die Analyse des Gesundheitszustands, -verhaltens und der Interessen an Maßnahmen der Gesundheitsförderung und Prävention sowie die dabei vorliegenden Unterschiede bei Studierenden der Erziehungswissenschaft und Medizin an der Universität Augsburg, um auf dieser Grundlage zukünftige spezifische Interventionen abzuleiten.

**Material und Methoden:**

Datenbasis bilden Online-Befragungen von Studierenden im Bachelor und Master der Erziehungswissenschaft (*N* = 163) und Humanmedizin (*N* = 100) an der Universität Augsburg im Wintersemester 2021/2022. Der Gesundheitszustand, das Gesundheitsverhalten sowie die Interessen an Themen, Formaten und Ideen für die Gesundheitsförderung im Setting Universität werden anhand von uni-, bi- und multivariaten Analysen dargestellt.

**Ergebnisse:**

Beide Studierendengruppen weisen eine verminderte psychische Gesundheit auf. Verglichen mit Medizinstudierenden weisen die befragten Erziehungswissenschaftsstudierenden höhere Risiken für ein depressives Syndrom, eine generalisierte Angststörung sowie körperliche Beschwerden auf. Etwa jede(r) dritte Befragte aus beiden Fächergruppen zeigt ein hohes Erschöpfungserleben als eine Subdimension von Burnout und einen riskanten Alkoholkonsum. Bei 65,6 % der Erziehungswissenschafts- und bei 41,0 % der Medizinstudierenden finden sich Hinweise auf eine internetbezogene Störung. Nahezu jede(r) zweite Studierende wünscht sich Kursangebote zu Bewegung sowie zu Entspannung/Stressbewältigung. Erziehungswissenschaftsstudierende zeigen einen größeren Bedarf an Angeboten zur psychischen Gesundheit als Medizinstudierende.

**Schlussfolgerung:**

Bei Studierenden der Erziehungswissenschaft und Medizin besteht ein hoher Bedarf an Maßnahmen zu psychischer Gesundheit, Alkohol- und Internetkonsum sowie Erschöpfung und Überforderungsgefühlen im Setting Universität. Bei der Umsetzung sollten Studiengangsspezifika beachtet und ein studentisches Gesundheitsmanagement vorangebracht werden, das auch Studierende weiterer Studiengänge berücksichtigt.

## Abstract (en)

**Background:**

Students in Germany are less likely to report good subjective health and more likely to have depressive symptoms than the 18- to 29-year-old general population.

**Objectives:**

The aim of the survey is to analyse health status, health behaviour and the topics of interest for health promotion and prevention measures as well as the differences present in this regard among students of education science and medicine at the University of Augsburg in order to derive future specific interventions.

**Materials and methods:**

The data were collected from online surveys of students in the bachelor and master of education science (*N* = 163) and human medicine (*N* = 100) at the University of Augsburg in the 2021/2022 winter term. Health status, health behaviour and interests in topics, formats and ideas for health promotion in the university setting are presented using uni-, bi- and multivariate analyses.

**Results:**

Both student groups show decreased mental health. Compared to medical students, the surveyed education science students show higher risks for depression, generalized anxiety disorder and physical complaints. About one in three respondents from both subject groups show a high experience of exhaustion as a subdimension of burnout and risky alcohol consumption. In 65.6% of education science and 41.0% of medical students there is evidence of an internet-related disorder. Almost every second student expressed interest in courses on physical exercise and relaxation/stress management. Education science students show a greater need for mental health services than medical students.

**Conclusion:**

Among students of education science and medical education, there is a high demand for preventive measures on issues with mental health, alcohol and internet consumption as well as exhaustion in the university setting. The implementation of these measures should consider the specifics of the study programs and promote a student health management that also takes into account students from other course of studies.

## Einleitung/Forschungsstand

Repräsentative Daten zum Gesundheitszustand wie auch zum Gesundheitsverhalten von Studierenden in Deutschland liefert eine Querschnittbefragung der Freien Universität Berlin aus dem Jahr 2017 [8], wonach Studierende seltener einen guten subjektiven Gesundheitszustand aufweisen und häufiger eine depressive Symptomatik angeben als die Gruppe der 18- bis 29-Jährigen in der Allgemeinbevölkerung [10]. Knapp ein Viertel der Studierenden in Deutschland berichtet in dieser Studie ein hohes Burnout-Erleben in den Ausprägungen Erschöpfung und Bedeutungsverlust [8].

Dieses erscheint zunächst überraschend, da Erhebungen zur Bevölkerungsgesundheit regelmäßig von einem Bildungsgradienten berichten, woraus abgeleitet werden könnte, dass Studierende häufiger eine gute subjektive Gesundheit und eher seltener eine depressive Symptomatik aufweisen als Personen aus der mittleren oder unteren Bildungsgruppe [10]. Doch bereits eine Befragung im Auftrag des Deutschen Studierendenwerks aus dem Jahr 2012 [21] berichtet über einen hohen Problemdruck in den Bereichen Erschöpfung/Überforderungsgefühle, psychosomatische Beschwerden, Ängste, Lern- und Leistungsstörungen, übermäßige Internetnutzung und depressive Verstimmung bei 30–50 % der Studierenden in Bachelorstudiengängen. Auch ein Studierendensurvey in Nordrhein-Westfalen aus den Jahren 2006/2007 [20] zeigt, dass das Wohlbefinden von knapp jeder/jedem zweiten Studierenden durch Zeitstress, Hektik im Hochschulgebäude, fehlenden Rückzugsmöglichkeiten und fehlendem Praxisbezug im Studium beeinträchtigt wird.

Studien zur psychischen Gesundheit wurden vielfach bei Medizinstudierenden (M-Studierende) durchgeführt, für die national wie international eine hohe Prävalenz von Depressivität [23, 24] und im Verlauf des Studiums eine zunehmende Inzidenz von Burnout [4, 5] und Depressivität [19] beschrieben ist. Erschens et al. [6] führen auf, dass M‑Studierende häufiger von Burnout betroffen sind als die gleichaltrige Allgemeinbevölkerung in tertiärer Ausbildung. Eine Metaanalyse von Puthran et al. aus dem Jahr 2016 [23] zeigt im Vergleich mit Studierenden anderer Studiengänge jedoch keine unterschiedlichen Chancenverhältnisse bezüglich der Prävalenz einer Depression. Grützmacher et al. [8] berichten auf Grundlage der 2017 durchgeführten deutschlandweiten Studierendenbefragung ein depressives Syndrom für 10,6 % der Studierenden aus der Fächergruppe Medizin/Gesundheitswissenschaften und für 18 % in der Fächergruppe Sozialwissenschaften/-wesen/Psychologie/Pädagogik. Im Geschlechtervergleich weisen männliche Studierende häufiger einen guten subjektiven Gesundheitszustand auf als weibliche Studierende, die häufiger ein depressives Syndrom, eine generalisierte Angststörung, körperliche Beschwerden oder ein hohes Burnout-Erleben aufweisen als Studenten [8].

Während der COVID-19-Pandemie („coronavirus disease 2019“) scheint sich der mentale Gesundheitszustand der Studierenden in Deutschland verschlechtert zu haben: So berichtet eine Querschnittbefragung von Studierenden der Universität Leipzig im Sommer 2020 einen Anteil von 37 % der Studierenden mit depressivem Syndrom [13]. Holm-Hadulla et al. [11] zeigen bei Heidelberger Studierenden, dass im Jahr 2021 31,1 % der M‑Studierenden ein depressives Syndrom und 16 % eine generalisierte Angststörung aufweisen, aus den Fachbereichen Wirtschaft und Sozialwissenschaften sind es 43,7 % bzw. 23,1 %. Während der Pandemie besteht ein signifikanter Zusammenhang zwischen dem Studienfach und dem Vorliegen eines depressiven Syndroms oder einer generalisierten Angststörung [11]. Geäußerte Beschwerden wie ein schlechter subjektiver Gesundheitszustand, Einsamkeit, depressive Verstimmungen, fehlender Antrieb und Motivation sind laut Holm-Hadulla et al. [11] größtenteils auf die Kontaktbeschränkungen im sozialen Bereich zurückzuführen. Hervorzuheben ist, dass M‑Studierenden aufgrund ihrer Tätigkeit im Gesundheitsbereich mehr soziale Interaktion möglich war und sie mehr Selbstwirksamkeit erfahren haben [11].

Gesundheitsförderliche Angebote wünschen sich die Studierenden v. a. zu den Themen Stressbewältigung, Depression, Prüfungsangst, Zeitmanagement, Sucht und Bewegung [12, 20]. An einigen Hochschulen in Deutschland bestehen bereits Initiativen zur Förderung der Gesundheit von Studierenden oder sogar ein studentisches Gesundheitsmanagement und eine regelmäßige Berichterstattung zur Studierendengesundheit [3, 16]. An der Universität Augsburg besteht bislang kein studentisches Gesundheitsmanagement mit einer regelmäßigen Gesundheitsberichterstattung über alle Studiengänge hinweg, ist jedoch seit Januar 2023 im Aufbau. An der medizinischen Fakultät werden regelmäßig längsschnittliche Daten zur Gesundheit der M‑Studierenden erhoben. Die vorliegende Erhebung erfolgte im Rahmen des interdisziplinären Augsburger Projekts „Gesundheitsförderung im Studium. Interdisziplinär studieren, interprofessionell handeln“ (G.i.S.), das für interessierte Studierende der Medizin (M) und Erziehungswissenschaft (EW) den Erwerb von Handlungskompetenz im Bereich Gesundheitsförderung und Prävention als Ziel definiert. Im Rahmen eines Wahlfachs können Studierende im Sinne des Peer-to-peer-Ansatzes Maßnahmen zur Gesundheitsförderung und Prävention für andere Studierende an der Universität planen, umsetzen oder evaluieren. Im Sinne des Public Health Action Cycle wurde zunächst die Bedarfs- bzw. Problemlage anhand folgender Forschungsfragen analysiert.

## Forschungsfragen

Welchen Gesundheitszustand und welches Gesundheitsverhalten weisen Studierende der Medizin und der Erziehungswissenschaft nach 2 Jahren COVID-19 Pandemie auf und liegen Unterschiede vor?

Welche Interessenslagen an Themen und Formaten der Gesundheitsförderung und Prävention im Setting Universität lassen sich bei Studierenden der Medizin und Erziehungswissenschaft nach 2 Jahren COVID-19-Pandemie feststellen?

## Methodik

### Datenbasis und Stichprobe

Datenbasis bildet eine Querschnittbefragung der immatrikulierten Studierenden der Studiengänge Bachelor und Master Erziehungswissenschaft sowie Medizin an der Universität Augsburg im Wintersemester 2021/2022. Die Verarbeitungstätigkeit wurde mit dem Datenschutzbeauftragten der Universität Augsburg abgestimmt. Die EW-Studierenden erhielten die Befragungseinladung inklusive Informationen zur Befragung sowie Datenschutzhinweisen via Hochschul-E-Mail-Adresse und wurden nach einer Woche an die Befragung erinnert. Zusätzlich haben Vertreter:innen der Studierendenschaft die Befragung beworben, indem sie über die Social-media-Plattform Instagram auf die Befragung aufmerksam machten sowie daran erinnerten. Die M‑Studierenden im ersten und dritten Fachsemester wurden persönlich in einem Seminar über die Befragung informiert und zur Teilnahme via QR-Code motiviert; im Anschluss erhielten sie eine E‑Mail mit den Zugangsdaten zur Befragung. Die M‑Studierenden im fünften Semester wurden ausschließlich via E‑Mail informiert. Der Studiengang Humanmedizin befindet sich noch im Aufbau. Insgesamt haben 163 Studierende der EW (22 %) und 100 Studierende der M (36,5 %) einen Online-Fragebogen vollständig ausgefüllt. Im Fragebogen werden u. a. die Items subjektiver Gesundheitszustand, depressives Syndrom, generalisierte Angststörung, körperliche Beschwerden, Stress, internetbezogene Störung, (gesundheitsförderliche) sportliche Aktivität, Alkoholkonsum, Burnout-Erleben und das Interesse an gesundheitsförderlichen Angeboten mit etablierten Skalen abgefragt, auf die im weiteren Verlauf noch näher eingegangen wird. Fast zeitgleich lief bei den M‑Studierenden die seit 2019 am Lehrstuhl für Medizindidaktik und Ausbildungsforschung etablierte längsschnittliche Online-Befragung ELMA (Experienced Learning Medicine Augsburg). Um eine doppelte Abfrage gleicher Items zu vermeiden, wird bei den M‑Studierenden bei dem Item Burnout-Erleben auf die Daten der ELMA-Befragung mit Angaben von 218 M-Studierenden zurückgegriffen. Für die Analysen werden alle Daten in einem gemeinsamen Datensatz verarbeitet.

Der Großteil der Befragten aus beiden Studiengängen ist zwischen 18 und 29 Jahre alt. Der Anteil der weiblichen Teilnehmenden liegt bei den EW-Studierenden bei 89,0 % und bei den M‑Studierenden bei 67,0 % bzw. 66,1 % (ELMA). Weitere Angaben zur Stichprobe können der Tab. 1 entnommen werden.

**Tab. 1:** Stichprobenbeschreibung (*N* & %)

	Befragung Forschungsprojekt G.i.S. Erziehungswissenschaft		Befragung Forschungsprojekt G.i.S. Humanmedizin		Befragung „ELMA“ Humanmedizin (Burnout-Erleben)	
	Häufigkeiten		Häufigkeiten		Häufigkeiten	
	*n*	%	*n*	%	*n*	%
*Studiengang*						
Erziehungswissenschaft	163	100	–	–	–	–
Humanmedizin	–	–	100	100	218	100
*Fachsemester*						
1	42	25,8	44	44,0	91	41,7
2	–	–	–	–	–	–
3	37	22,7	44	44,0	80	36,7
4	5	3,1	–	–	–	–
5	42	25,8	12	12,0	47	21,6
6	3	1,8	–	–	–	–
7	18	11,0	–	–	–	–
8–12	16	9,8	–	–	–	–
*Geschlecht*						
Weiblich	145	89,0	67	67,0	144	66,1
Männlich	16	9,8	33	33,0	74	33,9
Divers	2	1,2	–	–	–	–
*Alter*						
< 18 Jahre	–	–	–	–	1	0,5
18–29 Jahre	146	89,6	99	99,0	212	97,2
30–44 Jahre	14	8,6	1	1,0	5	2,3
45–64 Jahre	2	1,2	–	–	–	–
65+ Jahre	–	–	–	–	–	–
Systembedingt fehlend	1	0,6	–	–	–	–

Der Modellstudiengang Humanmedizin wird seit Oktober 2019 an der Universität Augsburg angeboten; die Zulassung erfolgt nur zum Wintersemester. Datengrundlage: Online-Befragungen im Studiengang Bachelor und Master Erziehungswissenschaft, Studiengang Humanmedizin im WS 21/22 & ELMA-Befragung Humanmedizin (Burnout-Erleben) WS 21/22

*ELMA* Experienced Learning Medicine Augsburg, *G.i.S.* Gesundheitsförderung im Studium. Interdisziplinär studieren, interprofessionell handeln

### Variablen- und Skalenbeschreibung

Der subjektive Gesundheitszustand wird mit der Frage „Wie bewerten Sie Ihren Gesundheitszustand im Allgemeinen?“ erfasst. In Anlehnung an die internationale Übereinkunft der Weltgesundheitsorganisation (WHO; [2]) bilden die Antwortmöglichkeiten „sehr gut“ und „gut“ einen guten subjektiven Gesundheitszustand sowie „mittelmäßig“, „schlecht“ und „sehr schlecht“ einen schlechten subjektiven Gesundheitszustand ab. Die Selbsteinschätzung des eigenen allgemeinen Gesundheitszustands entspricht näherungsweise dem objektiven Gesundheitszustand, es besteht ein messbarer Zusammenhang [28].

Ein depressives Syndrom und eine generalisierte Angststörung werden mit der Kurzversion des „patient health questionnaire 4“ (PHQ4) erfasst [17]. Für das Vorliegen eines depressiven Syndroms sowie einer generalisierten Angststörung wird je ein Summenscore aus zwei Aussagen gebildet und ein Cut-off bei ≥ 3 gesetzt [18].

In Anlehnung an Grützmacher et al. [8] wurde die Häufigkeit von verschiedenen körperlichen Beschwerden (z. B. „Magen-Darm-Beschwerden“ oder „Kopfschmerzen“) anhand von sieben Antwortmöglichkeiten von „nie“ (1) bis „jeden Tag“ (7) erfasst. Betrachtet werden die Studierenden, bei denen das jeweilige Symptom mindestens ein paar Mal im Monat (4–7) auftritt.

Die Häufigkeit des Auftretens von Stress wird, wie in einer Studie der Techniker Krankenkasse zum Thema Stress [25], mit der Frage „Wie oft fühlen Sie sich gestresst?“ und den Antwortmöglichkeiten „häufig“, „manchmal“, „selten“, „nie“ und „weiß nicht“ erhoben.

Das Vorliegen einer Internetbezogenen Störung wird mit der Short Compulsive Internet Use Scale (Short CIUS; [1]) mit Hilfe von fünf Fragen erfasst. Durch Bildung eines Summenindexes und eines Cut-offs bei ≥ 7 Punkten kann der Anteil mit einer internetbezogenen Störung bzw. mit einer riskanten/schädlichen/abhängigen Nutzung festgestellt werden [1].

Die wöchentliche sportliche Aktivität wird, wie in der Studie zur Gesundheit Erwachsener in Deutschland (DEGS1; [14]), mit zwei Fragen erfasst. Dadurch kann der Anteil ermittelt werden, der die Empfehlung der WHO von 2,5 h moderater Aktivität pro Woche erreicht.

Der (riskante) Alkoholkonsum wird, wie im bundesweiten Gesundheitsmonitoring des Robert Koch-Instituts, mit dem Instrument AUDIT‑C [26] abgefragt; danach liegt ab einem Punktewert von 4 bei Frauen und 5 bei Männern ein riskanter Alkoholkonsum vor.

Das Burnout-Erleben wird mit der deutschen Kurzversion des Maslach-Burnout-Inventar – Student-Survey (MBI-SS KV) erfasst. Von den jeweiligen Burnout-Subdimensionen Erschöpfung, Bedeutungsverlust und reduziertes Wirksamkeitserleben wird gesprochen, wenn der jeweilige Summenindex auf einer siebenstufigen Häufigkeitsskala von „nie“ (0) bis „täglich“ (6) ≥ 4 ist [8, 27].

**Abb. 1:**
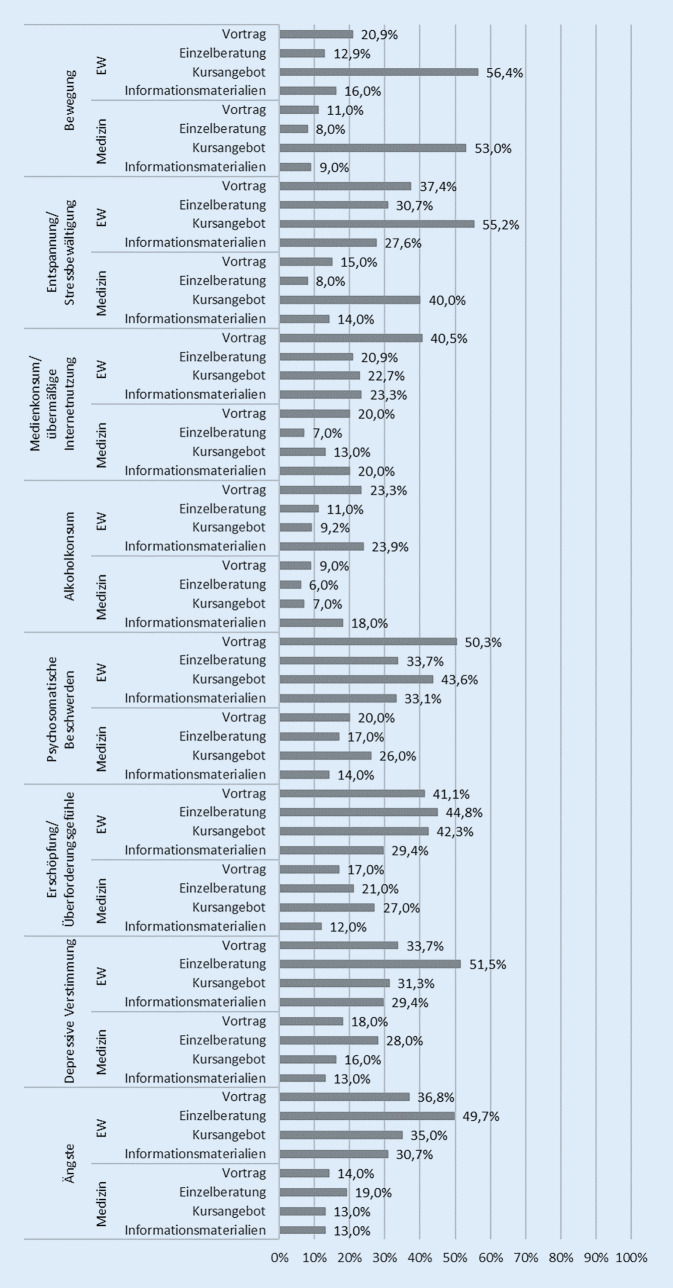
Interesse an Themen und Formaten der Gesundheitsförderung im Setting Universität, Studierende der Erziehungswissenschaft (*EW*) und der Medizin (Angaben in %; Mehrfachantworten möglich)

Die Abfrage des Interesses an gesundheitsförderlichen Angeboten im Setting Universität ist angelehnt an eine Studierendenbefragung aus Nordrhein-Westfalen aus dem Jahr 2007 [20] und ergänzt durch Erkenntnisse einer HISBUS-Befragung zur „Beratung von Bachelorstudierenden in Studium und Alltag“ [21] sowie eines Abschlussberichts zur „Studierendengesundheit in Stadt und Landkreis Würzburg (StuGeWü)“. Auf die Fragen „Haben Sie grundsätzlich Interesse an Angeboten der Gesundheitsförderung an der Hochschule?“ und „Wenn ja, an welchen Themen und Formaten der Gesundheitsförderung an der Hochschule haben Sie Interesse?“ können die Studierenden entlang verschiedener Themen (eine Auswahl ist in Abb. 1 dargestellt) ihr Interesse für die Formate Vortrag, Einzelberatung, Kursangebot oder Informationsmaterialien für ein oder mehrere Themen ankreuzen. Darüber hinaus besteht die Möglichkeit zur Freitexteingabe zur Frage: „Was würde ihr Wohlbefinden im Studium (und auf dem Campus) positiv beeinflussen?“

### Statistische Analysen

Die Interessen an verschiedenen Themen und Formaten der Gesundheitsförderung an der Universität werden anhand von Häufigkeiten in Prozent und nach dem Studiengang differenziert dargestellt. Die Antworten auf die Freitextfrage zur Steigerung des Wohlbefindens im Studium und auf dem Campus sind thematisch gruppiert und nach Häufigkeit der Nennungen gelistet.

Anhand von Kreuztabellen mit χ^2^-Signifikanzprüfung werden die Häufigkeiten für die Variablen subjektiver Gesundheitszustand, depressives Syndrom, generalisierte Angststörung, körperliche Beschwerden, internetbezogene Störung, (gesundheitsförderliche) sportliche Aktivität, Stress, Alkoholkonsum und die drei Subdimensionen von Burnout, differenziert nach dem Studiengang, berechnet. Mittels binär logistischer Regression werden die Chancenverhältnisse (OR) für die signifikanten Gesundheitsindikatoren von EW-Studierenden im Vergleich zu M‑Studierenden berechnet. Es werden zwei Modelle berechnet; im ersten Modell findet die Berechnung ohne Kontrollvariable statt, im zweiten Modell wird nach Geschlecht adjustiert.

Alle Auswertungen wurden mit dem Statistikprogramm IBM SPSS® Version 28 durchgeführt.

## Ergebnisse

Im Folgenden sind die Indikatoren des Gesundheitszustands und -verhaltens sowie Interessen an gesundheitsförderlichen Maßnahmen sowie die Unterschiede der beiden Studierendengruppen thematisch geordnet dargestellt. Von den befragten Studierenden geben 38,0 % (EW) und 29,0 % (M) einen schlechten subjektiven Gesundheitszustand an. Insgesamt äußern 84,7 % der EW-Studierenden und 71,0 % der M‑Studierenden Interesse an gesundheitsförderlichen Angeboten an der Universität.

### Bewegung und Entspannung/Stressbewältigung

Die wöchentliche Bewegungsempfehlung der WHO erreichen 84,0 % der EW-Studierenden und 75,0 % der M‑Studierenden nicht. Häufigen Stress geben 38,0 % aus dem Studiengang EW und 32,0 % aus dem Studiengang M an (Tab. 2). Es zeigen sich keine signifikanten Zusammenhänge zwischen dem Nicht-Erreichen der Bewegungsempfehlung sowie häufigem Stress und dem Studiengang.

**Tab. 2:** Häufigkeiten Indikatoren des Gesundheitszustands und -verhaltens, differenziert nach dem Studiengang (Angaben in % und *n* in Klammern, *p*-Wert; *N* = 263)

**–**	**Schlechter subjektiver Gesundheitszustand**	**Depressives Syndrom**	**Generalisierte Angststörung**	**Gesamt**
*Studiengang*	*p* = 0,135	*p* = 0,017*	*p* = 0,006**	–
Erziehungswissenschaft	38,0 (62)	32,5 (53)	33,7 (55)	62,0 (163)
Humanmedizin	29,0 (29)	19,0 (19)	18,0 (18)	38,0 (100)
*Gesamt*	34,6 (91)	27,4 (72)	27,8 (73)	100 (263)
**–**	**Herz-Kreislauf-Beschwerden**	**Magen-Darm-Beschwerden**	**Glieder‑, Schulter‑, Rücken‑, Nackenschmerzen**	–
*Studiengang*	*p* = 0,003**	*p* = 0,068	*p* = 0,005**	–
Erziehungswissenschaft	38,7 (63)	54,6 (89)	75,5 (123)	62,0 (163)
Humanmedizin	21 (21)	43,0 (43)	59,0 (59)	38,0 (100)
*Gesamt*	31,9 (84)	50,2 (132)	69,2 (182)	100 (263)
**–**	**Beeinträchtigtes Allgemeinbefinden**	**Anspannung**	**Kopfschmerzen**	–
*Studiengang*	*p* < 0,001***	*p* = 0,025*	*p* = 0,004**	–
Erziehungswissenschaft	68,1 (111)	49,1 (80)	57,1 (93)	62,0 (163)
Humanmedizin	42,0 (42)	35,0 (35)	39,0 (39)	38,0 (100)
*Gesamt*	58,2 (153)	43,7 (115)	50,2 (132)	100 (263)
**–**	**Internetbezogene Störung**	**Kein Erreichen der WHO-Bewegungsempfehlung** **(150** **min/Woche)**^**a**^	**Stress „häufig“**	–
*Studiengang*	*p* < 0,001***	*p* = 0,075	*p* = 0,321	–
Erziehungswissenschaft	65,6 (107)	84,0 (136)	38,0 (62)	62,0 (163)
Humanmedizin	41 (41)	75,0 (75)	32,0 (32)	38,0 (100)
*Gesamt*	56,3 (148)	80,5 (211)	35,7 (94)	100 (263)
**–**	**Riskanter Alkoholkonsum**	–	–	–
*Studiengang*	*p* = 0,154	–	–	–
Erziehungswissenschaft	38,7 (63)	–	–	62,0 (163)
Humanmedizin	30,0 (30)	–	–	38,0 (100)
*Gesamt*	35,4 (93)	–	–	100 (263)

Datengrundlage Online-Befragung Forschungsprojekt G.i.S. (Gesundheitsförderung im Studium. Interdisziplinär studieren, interprofessionell handeln) im Studiengang Erziehungswissenschaft und Humanmedizin, WS 21/22

*p* Signifikanzniveau: *p < 0,05; **p < 0,01; ***p < 0,001

^a^*N* = 262

Die befragten Studierenden wünschen sich v. a. Kursangebote zu Bewegung (EW = 56,4 %; M = 53,0 %) sowie zur Entspannung und Stressbewältigung (EW = 55,2 %; M = 40,0 %; Abb. 1).

### Medien- und Alkoholkonsum

Eine internetbezogene Störung bzw. eine riskante, schädliche oder abhängige Nutzung des Internets weisen 65,6 % der EW- und 41,0 % der M‑Studierenden auf. Es zeigt sich ein signifikanter Zusammenhang zwischen dem Vorliegen einer internetbezogenen Störung und dem Studiengang (*p* < 0,001). EW-Studierende haben verglichen mit M‑Studierenden und adjustiert nach Geschlecht (Modell 2), ein 3,33fach (*p* < 0,001; 95 %-Konfidenzintervall [KI]: 1,91–5,79) erhöhtes Risiko für eine internetbezogene Störung.

Mit Blick auf das Thema Medienkonsum und auf übermäßige Internetnutzung wünschen sich die Studierenden in erster Linie Maßnahmen in Form von Vorträgen (EW = 40,5 %; M = 20,0 %), gefolgt von Informationsmaterialien (EW = 23,3 %; M = 20,0 %; Abb. 1).

Einen riskanten Alkoholkonsum weisen 38,7 % der EW-Studierenden und 30,0 % der M‑Studierenden auf. Ein signifikanter Zusammenhang zwischen einem riskanten Alkoholkonsum und dem Studiengang besteht nicht.

Interesse artikulieren die befragten Studierenden v. a. an Informationsmaterialien (EW = 23,9 %; M = 18,0 %) und Vorträgen (EW = 23,3 %; M = 9,0 %) zum Thema Alkoholkonsum (Abb. 1).

### Körperliche Beschwerden

Es besteht ein signifikanter Zusammenhang zwischen körperlichen Beschwerden und dem Studiengang (Tab. 2): Beispielsweise geben 38,7 % der EW-Studierenden und 21,0 % der M‑Studierenden an, mindestens ein paar Mal im Monat an Herz-Kreislauf-Beschwerden zu leiden. Ein beeinträchtigtes Allgemeinbefinden nehmen 68,1 % der EW-Studierenden und 42,0 % der M‑Studierenden mindestens ein paar Mal im Monat wahr. Unter Kopfschmerzen leiden 57,1 % der EW- und 39,0 % der M‑Studierenden mindestens ein paar Mal im Monat. Adjustiert nach Geschlecht (Modell 2; Tab. 3) zeigt sich für Studierende der EW, verglichen mit Studierenden der M, ein um den Faktor 2,03 (*p* < 0,05; 95 %-KI: 1,12–3,69) erhöhtes Risiko für Herz-Kreislauf-Beschwerden, ein um den Faktor 2,64 (*p* < 0,001; 95 %-KI: 1,55–4,51) erhöhtes Risiko für ein beeinträchtigtes Allgemeinbefinden und ein um den Faktor 1,76 (*p* < 0,05; 95 %-KI: 1,04–2,99) erhöhtes Risiko für Kopfschmerzen.

**Tab. 3:** Binär-logistische Regressionsmodelle 1 und 2 für die Indikatoren des Gesundheitszustands und -verhaltens (OR und 95 %-KI; *N* = 263)

	Modell 1	Modell 2	Modell 1	Modell 2
	OR (95 %-KI)	OR (95 %-KI)	OR (95 %-KI)	OR (95 %-KI)
–	**Depressives Syndrom**		**Generalisierte Angststörung**	
*Studiengang*^*RK*^				
Erziehungswissenschaft	2,05 (1,13–3,73)*	2,08 (1,11–3,88)*	2,32 (1,27–4,25)**	2,14 (1,14–4,02)*
–	**Herz-Kreislauf-Beschwerden**		**Glieder‑, Schulter‑, Rücken‑, Nackenschmerzen**	
*Studiengang*^*RK*^				
Erziehungswissenschaft	2,37 (1,33–4,21)**	2,03 (1,12–3,69)*	2,14 (1,25–3,65)**	1,64 (0,93–2,89)
–	**Beeinträchtigtes Allgemeinbefinden**		**Anspannung**	
*Studiengang*^*RK*^				
Erziehungswissenschaft	2,95 (1,76–4,94)***	2,64 (1,55–4,51)***	1,79 (1,07–2,99)*	1,56 (0,91–2,67)
–	**Kopfschmerzen**		**Internetbezogene Störung**	
*Studiengang*^*RK*^				
Erziehungswissenschaft	2,08 (1,25–3,45)**	1,76 (1,04–2,99)*	2,75 (1,65–4,59)***	3,33 (1,91–5,79)***

Datengrundlage Online-Befragung Forschungsprojekt G.i.S. (Gesundheitsförderung im Studium. Interdisziplinär studieren, interprofessionell handeln) im Studiengang Erziehungswissenschaft und Humanmedizin, WS 21/22; Modell 1: Studiengang (RK: Medizin); Modell 2: Studiengang (RK: Medizin) unter Kontrolle von Geschlecht

*OR* Odds Ratio, *KI* Konfidenzintervall, *RK* Referenzkategorie

*p* Signifikanzniveau: ^*^p < 0,05; ^**^p < 0,01; ^***^p < 0,001

Angebote zum Thema psychosomatische Beschwerden wünschen sich die Studierenden vor allem in Form eines Vortrags (EW = 50,3 %; M = 20,0 %) oder Kurses (EW = 43,6 %; M = 26,0 %; Abb. 1).

### Burnout-Erleben

Ein hohes Erschöpfungserleben, eine Subdimension von Burnout zeigen 28,8 % der EW- und 32,6 % der M‑Studierenden. Die Subdimension reduziertes Wirksamkeitserleben zeigen 14,7 % der EW- und 10,6 % der M‑Studierenden. Bei der Subdimension Bedeutungsverlust zeigt sich ein höchst signifikanter Zusammenhang (*p* < 0,001) mit dem Studiengang (Tab. 4); 14,1 % der EW-Studierenden und 1,8 % der M‑Studierenden geben die Subdimension Bedeutungsverlust an. Für das Vorliegen von Bedeutungsverlust liegt für EW-Studierende, adjustiert nach Geschlecht (Modell 2), ein um das 11,21fache (*p* < 0,001; 95 %-KI: 3,55–35,45) erhöhtes Risiko vor, im Vergleich zu M‑Studierenden (Tab. 5).

**Tab. 4:** Häufigkeiten der Burnout-Subdimensionen Erschöpfung, Bedeutungsverlust und reduziertes Wirksamkeitserleben, differenziert nach dem Studiengang (Angaben in % und *n* in Klammern, *p*-Wert; *N* = 381)

	Burnout: Subdimension Erschöpfung	Burnout: Subdimension Bedeutungsverlust	Burnout: Subdimension reduziertes Wirksamkeitserleben	Gesamt
*Studiengang*	*p* = 0,435	*p* < 0,001***	*p* = 0,220	–
Erziehungswissenschaft	28,8 (47)	14,1 (23)	14,7 (24)	42,8 (163)
Humanmedizin	32,6 (71)	1,8 (4)	10,6 (23)	57,2 (218)
*Gesamt*	31,0 (118)	7,1 (27)	12,3 (47)	100 (381)

Datengrundlage Online-Befragung Forschungsprojekt G.i.S. (Gesundheitsförderung im Studium. Interdisziplinär studieren, interprofessionell handeln) im Studiengang Erziehungswissenschaft und ELMA-Befragung (Experienced Learning Medicine Augsburg) Humanmedizin, WS 21/22

*p* Signifikanzniveau: *p < 0,05; **p < 0,01; ***p < 0,001

**Tab. 5:** Binär-logistische Regressionsmodelle 1 und 2 für Burnout-Subdimension Bedeutungsverlust (OR und 95 %-KI; *N* = 381)

	Burnout: Subdimension Bedeutungsverlust	
	Modell 1	Modell 2
	OR (95 %-KI)	OR (95 %-KI)
*Studiengang*^*RK*^		
Erziehungswissenschaft	8,79 (2,98–25,96)***	11,21 (3,55–35,45)***

Datengrundlage Online-Befragung Forschungsprojekt G.i.S. (Gesundheitsförderung im Studium. Interdisziplinär studieren, interprofessionell handeln) im Studiengang Erziehungswissenschaft und ELMA-Befragung (Experienced Learning Medicine Augsburg) Humanmedizin, WS 21/22; Modell 1: Studiengang (RK: Medizin); Modell 2: Studiengang (RK: Medizin) unter Kontrolle von Geschlecht

*OR* Odds Ratio, *KI* Konfidenzintervall, *RK* Referenzkategorie

*p* Signifikanzniveau: ^***^p < 0,001

Zum Themenfeld Erschöpfung und Überforderungen wünschen sich die befragten Studierenden sowohl Kurse (EW = 42,3 %; M = 27,0 %), als auch Angebote in Form von Einzelberatung (EW = 44,8 %; M = 21,0 %). Auch besteht Interesse an Vorträgen zu diesem Themenkomplex (EW = 41,1 %; M = 17,0 %; Abb. 1).

### Depressives Syndrom und generalisierte Angststörung

Ein depressives Syndrom weisen 32,5 % der EW-Studierenden und 19,0 % der M‑Studierenden auf (Tab. 2). Zwischen dem Vorliegen eines depressiven Syndroms und dem Studiengang besteht ein signifikanter Zusammenhang (*p* = 0,017). Eine generalisierte Angststörung liegt bei 33,7 % der EW- und bei 18,0 % der M‑Studierenden vor; auch hier besteht ein signifikanter Zusammenhang mit dem Studiengang (*p* = 0,006). Studierende der EW weisen im Vergleich mit M‑Studierenden, adjustiert nach Geschlecht (Modell 2), ein 2,08fach (*p* < 0,05; 95 %-KI: 1,11–3,88) erhöhtes Risiko für ein depressives Syndrom und ein 2,14fach (*p* < 0,05; 95 %-KI: 1,14–4,02) erhöhtes Risiko für eine generalisierte Angststörung auf (Tab. 3).

Beim Thema depressive Verstimmung bzw. Ängste wünschen sich beide Studiengänge v. a. das Format einer Einzelberatung (EW = 51,5 % bzw. 49,7 %; M = 28,0 % bzw. 19,0 %; Abb. 1).

### Studentische Vorschläge für die Verbesserung des Wohlbefindens

Die Freitextfrage „Was könnte Ihr Wohlbefinden im Studium (und auf dem Campus) positiv beeinflussen?“ haben 35,6 % (*n* = 58) der befragten EW- und 21 % (*n* = 21) der M‑Studierenden beantwortet. Die häufigsten Nennungen der EW-Studierenden betreffen den Bereich der sozialen Beziehungen zu Kommiliton:innen wie auch zu Dozent:innen (z. B. „Kontakte/Freunde finden“ oder „Mehr Austausch mit Dozierenden“). Auch mehr Präsenzlehre und der Bereich Seminargestaltung (z. B. „weniger Referate“, „Projekte durchführen & planen“) werden häufig benannt. Bei den M‑Studierenden werden angenehm gestaltete Rückzugs- und Arbeitsräume (z. B. „Räume zum Zurückziehen für Pausen im Fakultätsgebäude“, „Steharbeitstische“ oder „Bilder, Poster, Farben etc.“), gefolgt von einer verbesserten Studienorganisation (z. B. „Genau definierte Lernziele/was wir alles wissen müssen, wie es geprüft wird“, oder „Online-stellen der Vorlesung, damit Fahrzeit zur Uni für Ausgleich zum Beispiel Sport genutzt werden kann“) genannt. Auch sie wünschen sich mehr soziale Kontakte (z. B. „Gruppenzugehörigkeit/nicht allein sein“, „Präsenzangebote wie Exkursionen oder Spieleabende“). An vierter Stelle wird in beiden Studiengängen explizit der aus Sicht der Studierenden zu hohe Workload genannt (z. B. „Weniger Workload, weniger Präsentationen, weniger Druck und Angst in Seminaren, wenn man es nicht schafft alle Arbeitsaufträge zu erledigen“).

## Diskussion

Ziel der Befragung war die Analyse des Gesundheitszustands und des Gesundheitsverhaltens von Studierenden sowie deren Interessen an Maßnahmen der Gesundheitsförderung und Prävention im Setting Universität. Hierbei wurden Studierende der Medizin sowie der EW in den (vergleichenden) Blick genommen. In Übereinstimmung mit bereits existierenden Untersuchungen [12, 20] zeigt sich auch in der vorliegenden Studie ein großer Bedarf an gesundheitsförderlichen Maßnahmen im Setting Universität wobei die vorliegende Befragung zeigen konnte, wie sich die Bedarfe je nach Studiengang unterscheiden.

Wie bereits in anderen Untersuchungen beschrieben [8], ist der Anteil der Studierenden mit einem riskanten Alkoholkonsum auch bei den hier befragten Studierenden hoch: Etwa jede/jeder dritte Studierende aus beiden Fächern weist einen riskanten Alkoholkonsum auf. Zur Auseinandersetzung mit ihrem Alkoholkonsum wünschen sich die befragten Studierenden v. a. Informationsmaterialien und Vorträge. Diese Maßnahmenformate empfehlen auch Laging et al. [15], wonach Studierende bei der Alkoholprävention vor allem Angebote wie Infobroschüren, Flyer, Plakate, anonyme web-basierte Angebote oder wissenschaftliche Vorträge gegenüber persönlichen Beratungen bevorzugen, da bei diesen Flexibilität und Anonymität gegeben sind.

Für rund zwei Drittel der befragten EW-Studierenden und zwei Fünftel der M‑Studierenden ist eine Intervention aufgrund einer vorliegenden internetbezogenen Störung angeraten, um das Abrutschen in eine pathologische Internetabhängigkeit zu verhindern [1]. Das erhöhte Risiko von EW-Studierenden für eine internetbezogene Störung, im Vergleich zu M‑Studierenden, könnte aufgrund einer Kompensation der enorm reduzierten persönlichen Kontakte im Zuge der pandemiebedingten Online-Lehre entstanden sein. Auffällig ist auch, dass v. a. die Unterfrage „Wie häufig gehen Sie ins Internet, wenn Sie sich niedergeschlagen fühlen?“ von über der Hälfte der EW-Studierenden mit „(sehr) häufig“ beantwortet wurde; demnach könnte die erhöhte Internetnutzung auch mit dem allgemein schlechter bewerteten psychischen Gesundheitszustand der EW-Studierenden im Vergleich zu den M‑Studierenden zusammenhängen. Die zentrale Studienberatung der Universität Augsburg bietet bereits Beratung zum Thema Medien- und Substanzkonsum an. Aufgrund der Befragungsergebnisse sowie den Studienergebnissen von Laging et al. [15] sollte das Angebotsformat auf niedrigschwellige Vorträge und Informationsmaterialien ausgeweitet werden, um Studierende besser zu erreichen.

Ebenso zeigen EW-Studierende erhöhte Risiken für das Vorliegen von körperlichen Beschwerden, verglichen mit M‑Studierenden. Eine mögliche Erklärung könnte sein, dass Studierende der EW während der COVID-19-Pandemie 2 Jahre größtenteils in der Online-Lehre verbracht haben, wohingegen M‑Studierende nie vollständig im Distanzunterricht waren, da insbesondere der Unterricht an Patient:innen weitestgehend regelhaft stattgefunden hat und soziale Kontakte zu Mitstudierenden und Dozierenden fortbestanden. Wie einleitend berichtet, zeigt Holm-Hadulla et al. [11], dass ein verschlechterter Gesundheitszustand v. a. auf Kontaktbeschränkungen im sozialen Bereich zurückzuführen ist. Grützmacher et al. [8] beschreiben, dass sich belastende und stressreiche Lebensumstände von Studierenden im Zuge von Somatisierungsprozessen in unterschiedlichen physischen Beschwerden ausdrücken können. Demnach ist es angeraten, neben dem von den Studierenden gewünschten Vortrags- und Kursangebot zu psychosomatischen Beschwerden, auch die Belastungs- und Stresssituationen im Studium im Sinne der Verhältnisprävention in den Blick zu nehmen.

In der vorliegenden Befragung bestätigt sich, dass Studierende der EW während der COVID-19-Pandemie häufiger ein depressives Syndrom und eine generalisierte Angststörung angeben als Studierende der Medizin [11, 13]. Übereinstimmend mit vergangenen Befragungen [21] besteht auch in der vorliegenden Erhebung ein hoher Beratungsbedarf bezüglich depressiver Verstimmung, welcher bei den befragten EW-Studierenden in Augsburg noch einmal deutlich erhöht ist: Jede(r) zweite EW-Studierende, aber auch ein großer Anteil der M‑Studierenden, äußert Interesse an Einzelberatung zu depressiver Verstimmung und Ängsten. Viele Universitäten und Studierendenwerke bieten bereits psychologische Beratung und Unterstützung bei psychischen Beschwerden an. Dies gilt auch für die Augsburger Universität und das hiesige Studierendenwerk. Insofern stellt sich weiterführend die Frage, ob das Angebot bei den Studierenden ausreichend bekannt ist bzw. welche Hürden einer Inanspruchnahme im Wege stehen.

Dass M‑Studierende weniger Bedeutungsverlust im Studium aufweisen als EW-Studierende und, verglichen mit Ergebnissen aus einer repräsentativen Studierendenbefragung von 2017 [8], sogar etwas besser abschneiden als vor der Pandemie könnte daran liegen, dass sowohl die Bedeutsamkeit des Arztberufes als auch die aktive Einbindung von M‑Studierenden in die Gesundheitsversorgung vor allem zu Beginn der Pandemie in der Gesellschaft allzeit präsent waren. Eine longitudinale Studie von Polujanski et al. [22] zeigt für die Augsburger M‑Studierenden eine Zunahme des Empfindens von Stolz während des ersten Online-Semesters in der Pandemie im Jahr 2020. Ebenso kann ein anhaltender Erschöpfungszustand aufgrund von Studienanforderungen, welche das Leistungsvermögen von Studierenden übersteigen, und fehlenden Erholungsmöglichkeiten zu einem Bedeutungsverlust sowie weiterführend zu einer Reduzierung des Wirksamkeitserlebens führen [8, 27]. Das Initialsymptom von Burnout, Erschöpfung, berichtet fast jeder dritte Studierende in beiden Studiengängen. Als wesentlichen Verbesserungspunkt für das Wohlbefinden im Studium nennen die Studierenden eine Anpassung des jeweiligen Workloads. Auch besteht seitens der Studierenden eine hohe Nachfrage nach Kurs‑, Vortrags- oder Beratungsangeboten zum Thema Erschöpfung/Überforderungsgefühle. Die Studienergebnisse legen nahe, dass Burnout-Prävention im Setting Universität nur mit einer Kombination aus verhaltens- und verhältnisbezogenen Maßnahmen gelingen kann.

Die aus den Daten der Befragung abgeleiteten Maßnahmenvorschläge können an verschiedenen Stellen an der Universität, z. B. der Studierendenberatung sowie dem Projekt G.i.S. im Peer-to-peer-Ansatz und im Rahmen des sich im Aufbau befindlichen Studentischen Gesundheitsmanagements der Universität Augsburg inklusive eines Monitorings der Studierendengesundheit [7] aufgegriffen und umgesetzt werden. Insbesondere der Peer-to-peer-Ansatz soll mittelfristig im Rahmen eines Wahlfaches als fester Baustein des studentischen Gesundheitsmanagements implementiert und verstetigt werden. Die Studierenden werden auf diese Weise in die Umsetzung wie auch kontinuierliche Weiterentwicklung von Maßnahmen zur Gesundheitsförderung auf dem Campus eingebunden.

Die vorliegende, EW- und M‑Studierende vergleichende Untersuchung hat deutlich gemacht, dass eine Gesundheitsberichtserstattung nach Studiengängen zu differenzieren hat. Dies wäre wiederum die Voraussetzung, um zielgruppengenaue Angebote zur Prävention und Gesundheitsförderung im Setting Universität etablieren zu können. Eine universitäre Gesundheitsberichterstattung für Studierende sollte, neben den abgefragten Indikatoren wie Gesundheitszustand, -verhalten und Interessen, auch eine differenzierte Erhebung von studienbezogenen Anforderungen, wie z. B. Zeitaufwand für Veranstaltungsbesuche oder Vor- und Nachbereitung sowie studienbezogene Ressourcen wie z. B. Handlungs- und Entscheidungsspielräume mit einbeziehen [9]. Dies wäre eine wichtige Basis, insbesondere für nachhaltige verhältnisbezogene Maßnahmen.

## Limitationen

Es ist möglich, dass sich an der Befragung vorwiegend Studierende beteiligt haben, die insgesamt am Thema Gesundheit interessiert sind, wodurch die Befragungsergebnisse ein verzerrtes Bild wiedergeben würden. Aufgrund des Querschnittdesigns sind keine kausalen Rückschlüsse möglich. Ebenso wurde in der vorliegenden Erhebung der sozioökonomische Status der Studierenden, ein wichtiges Einflussparameter auf Gesundheitszustand und -verhalten, nicht berücksichtigt.

## Fazit für die Praxis

- In beiden Studiengängen weisen viele Studierende einen riskanten Alkohol- und Internetkonsum sowie eine hohe Erschöpfung auf. Vor allem Erziehungswissenschaftsstudierende weisen nach 2 Jahren COVID-19-Pandemie („coronavirus disease 2019“) hohe Raten an depressivem Syndrom, generalisierter Angststörung und psychosomatischen Beschwerden auf.
- Studierende zeigen Interesse an gesundheitsförderlichen Angeboten zu psychischer Gesundheit. Dies scheint laut der vorliegenden Befragung insbesondere für Studiengänge mit pandemiebedingter Online-Lehre zu gelten. Bei der Entwicklung und Umsetzung von Maßnahmen speziell für diese Studierenden ist auf Partizipation und Empowerment zu achten.
- Die Befragungsergebnisse legen nicht zuletzt ein Überdenken bestimmter, von den Studierenden als belastend empfundenen Studiengangsstrukturen und Workload-Verteilungen nahe.
- Um wirksame verhaltens- als auch verhältnispräventive Maßnahmen der Gesundheitsförderung und Prävention im Setting Universität anzugehen, ist der Aufbau eines universitären Gesundheitsmanagements mit einem studiengangspezifischen Monitoring zu forcieren.
